# Bio-Benchmarking of Electronic Nose Sensors

**DOI:** 10.1371/journal.pone.0006406

**Published:** 2009-07-29

**Authors:** Amalia Z. Berna, Alisha R. Anderson, Stephen C. Trowell

**Affiliations:** CSIRO Entomology and CSIRO Food Futures Flagship, Canberra, Australian Capital Territory, Australia; University of California Davis, United States of America

## Abstract

**Background:**

Electronic noses, E-Noses, are instruments designed to reproduce the performance of animal noses or antennae but generally they cannot match the discriminating power of the biological original and have, therefore, been of limited utility. The manner in which odorant space is sampled is a critical factor in the performance of all noses but so far it has been described in detail only for the fly antenna.

**Methodology:**

Here we describe how a set of metal oxide (MOx) E-Nose sensors, which is the most commonly used type, samples odorant space and compare it with what is known about fly odorant receptors (ORs).

**Principal Findings:**

Compared with a fly's odorant receptors, MOx sensors from an electronic nose are on average more narrowly tuned but much more highly correlated with each other. A set of insect ORs can therefore sample broader regions of odorant space independently and redundantly than an equivalent number of MOx sensors. The comparison also highlights some important questions about the molecular nature of fly ORs.

**Conclusions:**

The comparative approach generates practical learnings that may be taken up by solid-state physicists or engineers in designing new solid-state electronic nose sensors. It also potentially deepens our understanding of the performance of the biological system.

## Introduction

Electronic noses, E-Noses, incorporate an array of chemical sensors of different specificities, which simultaneously respond to the volatile chemicals present in a gas sample. The two main components of an electronic nose are the sensing system and the automated pattern recognition system. The sensing system can be an array of gas sensors or it can be a single device. Gas sensors, based on chemical sensitivity of semiconducting metal oxides, are readily available commercially and have been more widely used to make arrays for odor measurements than any other single class of gas sensor [1]. They are characterised by a relatively fast response, typically less than 10 seconds, and they have high sensitivity to a range of organic vapours. Metal oxide (MOx) sensors consist of a metal-oxide semiconducting film (e.g. SnO_2_, TiO_2_, ZnO, ZrO_2_) coated onto a ceramic substrate (e.g. alumina). Most often the device also contains a heating element. Oxygen from the air is dissolved in the semiconductors' lattice, setting its electrical resistance to a background level. During measurement, volatiles are adsorbed at the surface of the semiconductor where they react with the dissolved oxygen species causing a further modification of the resistance of the device [2].

A number of types of E-Noses, which are based on different sensing technologies [3], are available commercially, however they have not been widely adopted, in large part because they perform poorly in some real-world discrimination tasks [4]. In order to improve upon existing E-Nose sensors, it would be helpful to define and, where possible, quantify the gap between their performance and the performance of a “gold standard”. Biological odorant receptors (ORs) are potentially useful references for E-Nose sensor but, until recently, more was known about the pathways that process olfactory information than about the function of ORs. However, following recent descriptions of the molecular physiology of one class of dORs [5], [6] and detailed characterization of the molecular receptive range of a subset of these receptors [7], it is now possible to compare the responses of a set of technical sensors with those derived by evolution. For example, using Hallem's dataset [7], Haddad et al. [8] trained a 16 sensor electronic nose to predict the likely responses of the rat I7 OR to other odorants. Here, we use the odor space defined by the set of odorants selected by Hallem to investigate the sensitivity, tuning and independence of 12 MOx sensors from an electronic nose. This type of sensors was chosen because of the advantages rehearsed above (high sensitivity, commercially available, fast response). The E-Nose sensors are compared with 24 Drosophila general odorant receptors, using Hallem's own data set [7]. Attempting to replicate the performance of a biological system in an engineered instrument would deepen our understanding of the underlying physiology and may also lead to technological improvements in instruments designed to perform similar tasks.

## Results and Discussion

### Insect ORs have substantially broader odorant specificity than MOx sensors

**Figure 1:**
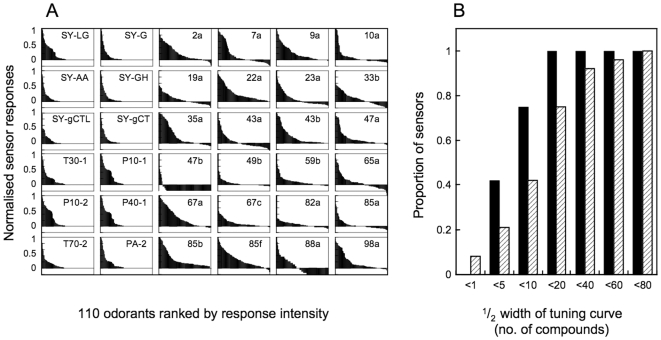
Odorant tuning curves and half-widths of metal oxide (MOx) sensors and Drosophila odorant receptors (dORs). A. MOx sensors (first two columns) and dORs (columns 3–6). Responses for all 110 odorants were obtained at, or scaled to, a dilution of 1/100 and normalised to a maximum response of 1.0 for each of the receptors/sensors. Drosophila data are recalculated from Hallem et al. [7] and electronic nose data from Table S1. The odorant tuning curves of MOx sensors have, on average, one third the half-width of dORs. Many of the odorants provoke inhibitory (negative) responses from a number of the dORs. These have been truncated. B. Cumulative histogram showing the distribution of receptor/sensor half-widths for odorant tuning. MOx sensors: cross-hatched bars. dORs: solid bars.

Using a panel of 110 odorants likely to be encountered in the natural habitat of the fly, Hallem et al. [7] demonstrated that the odorant tuning of fly receptors ranges from relatively narrow to very broad. We compared the fly's OR tuning curves, using Hallem's own data, with the tuning of the twelve MOx sensors from a Fox 3000 electronic nose (Alpha-MOS, Toulouse). For the fly, all liquid odorants were used at a nominal dilution of 1/100 and solid odorants at 1/50 [9]. This approach is not possible with the electronic nose because the sensitivity of the sensors varies profoundly according to the chemical class of odorants. Furthermore concentrations of odorants sufficiently high to elicit a near-saturated electronic response may cause permanent damage to E-Nose sensors. Therefore we established a safe working dilution for each of the twelve broad chemical classes (see Methods) prior to conducting the test. Responses were subsequently scaled to a constant dilution of 1/100 (Table S1). The necessity for such a procedure itself indicates a substantial difference between biological ORs and MOx sensors. As previously demonstrated [7] at 1/100 dilution, most of the fly ORs tested are excited or inhibited by the majority of the test compounds whereas the MOx sensors respond to 50% or fewer of the compounds at that dilution factor (Figure 1A). The mean half width of the tuning curve for the set of fly ORs is 14±13 compounds and for the MOx sensors the mean half width is 6±4 compounds (Figure 1B).

As would be expected, lowering the concentrations of odorants is found to reduce the number of odorants that elicit strong responses, particularly from broadly tuned receptors [7]. A similar phenomenon applies to MOx sensors. In this light, it is important to note that the data sets that we compared both related to nominal 1/100 dilutions of odorants. Vapour pressure is the same for any given odorant regardless of which system it is tested on. Therefore for each odorant tested, the actual concentration at the sensor surface cannot be higher for the Drosophila OR (dOR) than for the MOx sensor. Indeed, it is likely to be somewhat lower in the fly because of the non-equilibrium nature of the headspace delivery in that system. Therefore, if any bias was introduced in the comparison of tuning breadth, its effect would be to underestimate the breadth of the tuning curves of the fly ORs relative to the MOx sensors.

It has long been proposed [10] that an optimal chemical sensor array would utilise sensors of broad and overlapping analyte sensitivities. Metal oxide sensors, which themselves have no basis for chemical specificity other than redox potential, might be expected to fit this requirement perfectly. What the comparison shows is that the MOx sensors are nowhere near as broadly tuned as biological ORs despite the general, and generally deserved, reputation of bioreceptors for exquisite ligand specificity.

The relative promiscuity of fly ORs for odorants can be illustrated by reference to classical “lock and key” membrane receptors such as G-protein coupled receptors (GPCRs). Whereas Hallem et al. [7] found that many of the fly ORs respond to the majority of the odorants tested, screening for classical GPCR ligands with targeted libraries typically yields hit rates of less than 0.01% [11]. In this context, even the more selective of the ORs should be thought of as having very broad tuning compared with conventional pharmacological receptors. GPCRs and the class of fly ORs discussed here are both seven-transmembrane proteins, albeit of distinct evolutionary origins. However, the profoundly different ligand-tuning characteristics of the ORs, implies that their ligand binding mechanism would be novel and substantially different from that of most described bioreceptors.

### Independence of sensors

**Figure 2:**
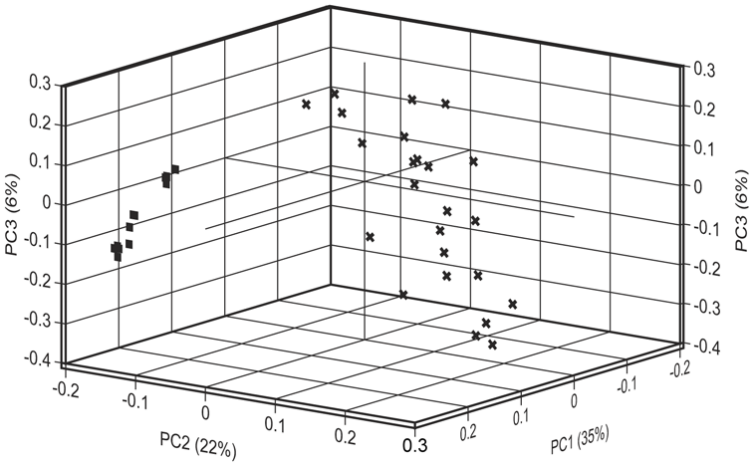
A comparison of the independence of MOx sensors and dORs in the same odorant space. Loading plot showing the first three principal components from a PCA analysis of the responses of dORs (crosses) and MOx sensors (squares) to 110 odorants. All odorants were tested at or scaled to a 1/100 dilution. The Pearson pairwise correlation coefficients between MOx or dOR pairs are show in Table S2.

The concept that the discriminating power of an electronic nose depends on the independence amongst its sensors, i.e. inversely on their redundancy or cross-correlation, is well known [12], [13], [14]. Hallem et al. [7] used principal components analysis of the data obtained with their test set to define and visualise the odorant space sampled by a subset of dORs. We ran principal components analysis (PCA) on the responses of both dORs and MOx sensors to the set of 110 test compounds first used by Hallem et al. [7]. This allowed us to compare the positioning of both types of sensors within the same odorant space and to compare between the average levels of correlation among sensors of the same type. As previously reported [7], the dORs are distributed relatively widely throughout the odorant space (Figure 2). In contrast, the 12 MOx sensors cluster together in two narrowly constrained regions of odorant space, indicating that the responses of the MOx sensors are highly correlated.

**Figure 3:**
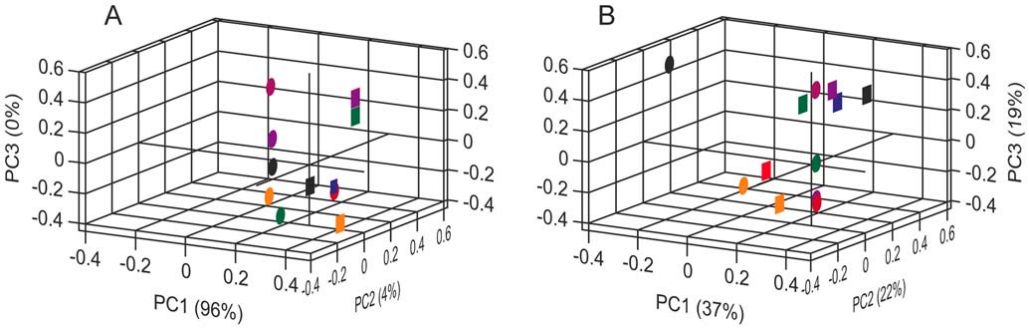
PCA loading plots for the responses of MOx sensors (A) and dORs (B) to 21 esters. The responses to all esters (compounds 22-42, Table S3) were tested at or scaled to 1/100 dilution and corrected to equivalent concentrations using vapour pressure data. The 12 dORs are those asterisked in Table S4 and were chosen to represent a full range of tuning half-widths. Pearson pairwise correlation coefficients between MOx or dOR pairs are given in Table S5.

To compare the sensor correlations we derived the pairwise Pearson correlation coefficients between all pairs within both classes of sensors (Table S2). For dORs, only five of the 276 possible sensor pairings were highly correlated (≥0.7 [15]) and the mean correlation was 0.24±0.28, whereas for the twelve MOx sensors all 66 of the possible sensor pairings were highly correlated with a mean correlation of 0.89±0.08. We next compared the independence of MOx sensors and dORs within a representative sub-region of odorant space defined by 21 esters. Esters form a chemical class of environmental significance for Drosophila and are also strong stimuli for MOx sensors. We selected only esters for which we had reliable vapour pressure information (Table S3) so that we could correct Hallem's data to minimise variation from this source [16], [17]. We also eliminated any bias due to different numbers of sensors by choosing a representative set of 12 dORs, spanning the full range of tuning curves (Table S4). Within the ester sub-region of odorant space, the 12 dORs (Figure 3B) were substantially more widely distributed than the MOx sensors (Figure 3A). Furthermore, we observed no increase in the correlations between pairs of dORs (Table S5; mean Pearson correlation = 0.12±0.4). The correlation among MOx sensor correlations was not significantly reduced (mean Pearson correlation = 0.77±0.25).

We are unaware of any prior use of quantitative statistics to define the levels of correlation amongst a set of odorant sensors. This is potentially a powerful approach, particularly if used in conjunction with a standard odorant set, which will assist in the selection and improvement of sensor technologies.

### Basis of compound discrimination

**Figure 4 pone-0006406-g004:**
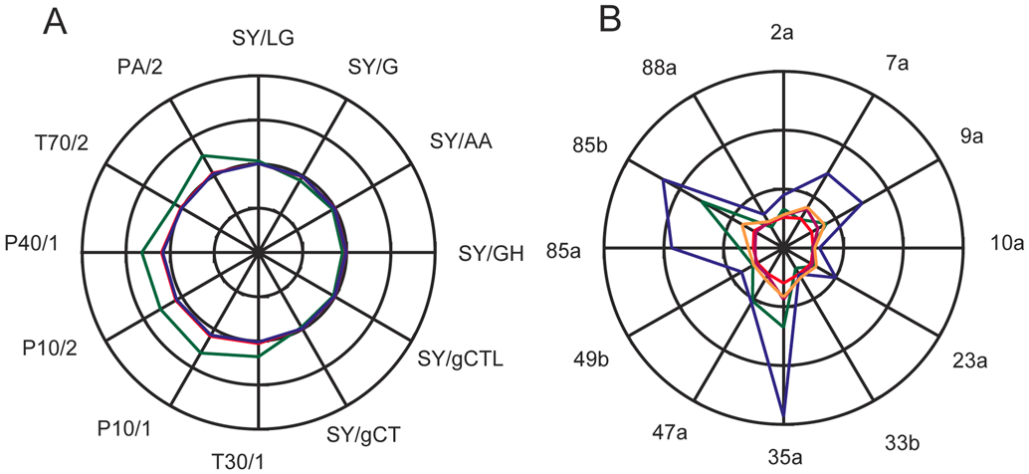
Radar plots for responses of the MOx sensors (A) and dOR sensors (B) to five alcohols. The scales represent full signal amplitude for Drosophila of −50 to +250 spikes per second for dORs and −1 to +1 arbitrary resistance units for MOx sensors. The five alcohols were: 1-pentanol (orange), 1-hexanol (blue), 3-methylbutanol (magenta), Z2-hexenol (red), 1-octen-3-ol (green), all at 1.4×10^−10^ M.

In relation to an electronic nose, Stetter et al. [12] wrote: “it is a bit frightening and unsettling to the analyst that the source of the chemical information is still so non-chemically specific.” Consistent with this we found, for example, that the pattern of MOx sensor responses for the alcohols: 1-pentanol, 1-hexanol, 3-methylbutanol, Z2-hexenol and 1-octen-3-ol are remarkably similar (Figure 4A), with only 1-octen-3-ol deviating from the general response envelope. However, the same set of alcohols varies enormously in the strength of responses they provoke from a set of 12 dORs (Figure 4B). Similar results were observed for other chemical classes (not shown).

**Figure 5:**
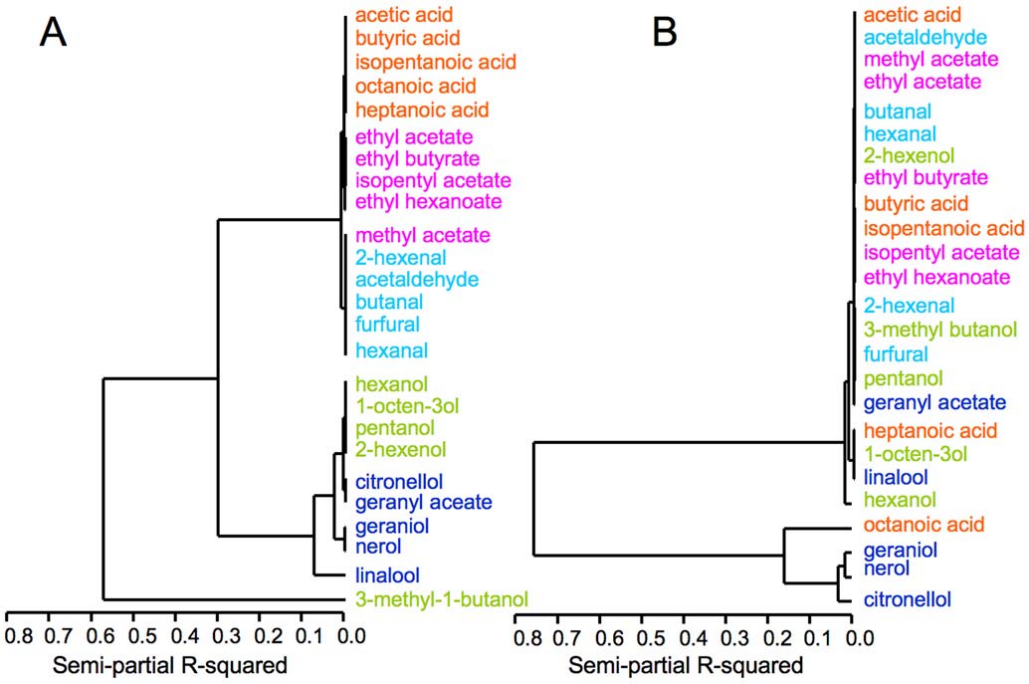
Cluster analysis for responses of MOx sensors (A) and dORs (B) to 25 compounds from five chemical classes. All responses are scaled to constant concentration: 4.20×10^−8^ M for MOx sensors, unknown for dORs. The chemical classes were: carboxylic acids (orange), alcohols (green), aldehydes (light blue), esters (maroon), terpenoids (dark blue).

To investigate this difference further, we performed cluster analysis (Figure 5A) for a set of 25 compounds (42 nM) from five chemical classes using MOx sensor data. We observed three statistically significant clusters corresponding to carbonyls (esters, aldehydes and carboxylic acid); 3-methyl butanol and several non-carbonyls; all other alcohols and terpenes. The clustering was broadly congruent with chemical functionality. A representative set of 12 dORs, also generated three clusters with significantly different semi-partial R^2^ values (Figure 5B). Three terpenes formed one cluster, octanoic acid a second and the other 21 compounds a third cluster. We could detect no statistically significant correlation with any external molecular characteristics, notwithstanding a weak association with mean molecular weight. Therefore, discrimination by odorant receptors, although robust, was based on subtle, undefined and highly compound-specific features of individual odorant molecules. The dissociation of the dOR-generated clusters from conventional molecular descriptors is a necessary corollary of the sensors being independent of each other.

These findings demonstrate that, if concentrations are standardised, E-Nose sensors can discriminate among chemical classes but have little capacity to discriminate amongst compounds with the same chemical functionality. The dependence of the MOx signal in part on chemical class or functionality is consistent with the working principle of this type of sensor, which is on-surface oxidation of analytes using molecular oxygen [1]. Oxidation of molecules sharing chemical functionality is likely to generate more similar redox potentials than would oxidation of molecules with different chemical functionality. Guerrieri et al. [16] proposed that the olfactory space of the honeybee could be defined “with functional group and carbon-chain length as inner dimensions”, which is not seen here for Drosophila. However, Guerreri et al. used a 16 compound test set, which was tightly constrained in chain length and functional group. Although we cannot exclude a species difference, we believe it is more likely that simple associations between olfactory classification and chemical structure break down over the larger distances in odorant space defined by the 110 compound test set used here.

### Comparison of the absolute sensitivities of dORs and MOx sensors

**Figure 6:**
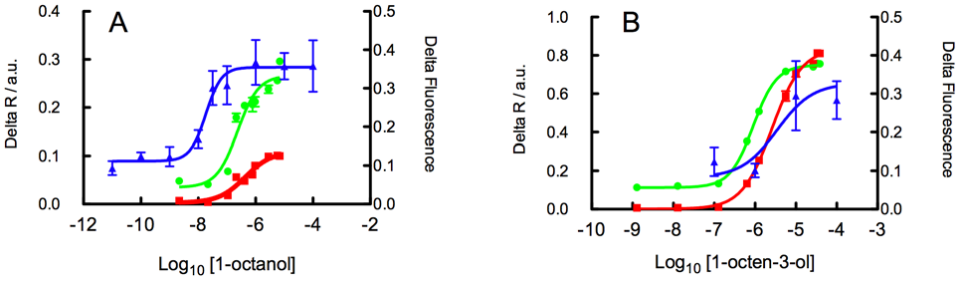
A comparison between the absolute sensitivities of MOx sensors (PA/2, SY/GH) and dOR35a. Log-concentration response curves show the sensitivity of dOR35a (blue), PA/2 (green) and SY/GH (red) to 1-octanol (A) and 1-octen-3-ol (B). MOx sensor responses are measured as fractional changes in resistance. dOR35a responses were recorded by measuring calcium-stimulated Fluo4 fluorescence intensity for receptors transiently expressed in Sf9 cells. Error bars represent the standard errors of the means and, for the MOx sensors, these are too small to be visualised.

One of the attractive features of MOx sensors is their high sensitivity to volatile odorants [1], which can confer a detection threshold comparable to that of humans, at least for some volatiles [4]. However, depending on the receptor and the volatile, the detection limits of biological receptors vary widely. In order to compare the sensitivities of different types of sensors it is necessary to relate their responses to known concentrations of the same odorants. When recording from the living fly, limitations of the stimulus presentation generally preclude knowledge of odorant concentrations. It is common practice to use standard dilutions of a stock odorant solution *in lieu* of defined absolute concentrations. Concentrations at the receptor vary depending on the vapour pressure of the odorant and the degree of extra dilution introduced in the airflow system and/or in diffusing from the insect cuticle to the receptor. The alternative approach pursued here, heterologous functional expression of insect ORs, permits known concentrations of odorants to contact the receptors in the liquid phase and enables direct measurements of OR sensitivity [18]. Measurements with the electronic nose are more straightforward because a predetermined concentration of odorant can be presented to the sensors. However, as far as we can ascertain, sensitivity of MOx sensors has not previously been stated in terms of an EC_50_ value at the sensor surface. Instead a limit of detection is determined and generally this relates to the level of analyte in the sample. The latter approach is ideal for determining the utility of MOx sensors for a particular task, however it is sub optimal for comparing the absolute sensitivities of different types of sensors. In the past, therefore, it has not been possible to make a rigorous comparison of the sensitivities of MOx sensors and biological ORs. In this study we have directly compared the EC_50_ values of MOx sensors. We surveyed the responses of all sensors to 89 compounds from the 110 odorant test panel for which we could obtain vapour pressure data. We selected the dOR35a receptor and MOx sensors PA/2 and SY/GH from a Fox 3000 electronic nose as representative receptors to perform a rigorous comparison because these sensors respond strongly to some of the same compounds. dOR35a is slightly unusual in that it is the only member of its class known to be expressed in one of the coelonic sensilla, which otherwise house a new class of variant ionotropic receptors [19]. In all other respects, dOR35a behaves as a normal member of its class. 1-octanol was selected as a test odorant because it gives the highest vapour pressure-adjusted response for dOR35a, generating an EC_50_≈23 nM for dOR35a (Figure 6A, Table 1). This compound was also detected as sensitively as any other by the MOx sensors (data not shown). Nevertheless, MOx EC_50_ values were 0.2–0.4 µM i.e. 10–20 fold higher than for dOR35a. 1-octen-3-ol was also selected, as representative of compounds that generate moderate responses in both types of sensors (Figure 6B, Table 1), in both the dOR35a and MOx sensors and elicited EC_50_ responses in the low micromolar range from both types of sensor. The EC_50_ values observed here represent a conservative upper limit for insect ORs. Using the same assay conditions, certain Drosophila [18] and lepidopteran [20], [21] OR/odorant combinations generate EC_50_ values as low as 14–16 pM to some odorants. It seems that, although the affinities of this group of MOx sensors for odorants overlap the working range for ORs, the affinities of some ORs may extend to at least four log units more sensitive than for the MOx sensors considered here. However, if the comparison were made in terms of limits of detection the relative sensitivity of the MOx sensors would be substantially better, due to their very low noise levels (Figure 6).

**Table 1:** EC_50_ and Hill coefficients derived from fitting log-concentration response curves for MOx sensors PA/2 and SY/GH and dOR35a.

Compound		dOr35a	PA/2	SY/GH
1-octanol	EC_50_ (nM) Hill Slope	22.7 (12.6–41)* 1.19±0.22*	233 (157–348) 1.19±0.27	434 (268–702) 0.935±0.22
1-octen-3-ol	EC_50_ (nM) Hill Slope	3,455 (700–17,000) n.d.	900 (854–950) 1.52±0.07	2,590 (2,290–2,930) 1.18±0.06

* 95% confidence limits for EC_50_ values and standard deviations for the Hill coefficients.

### Relative sampling of odorant space by MOx and OR sensors

**Figure 7:**
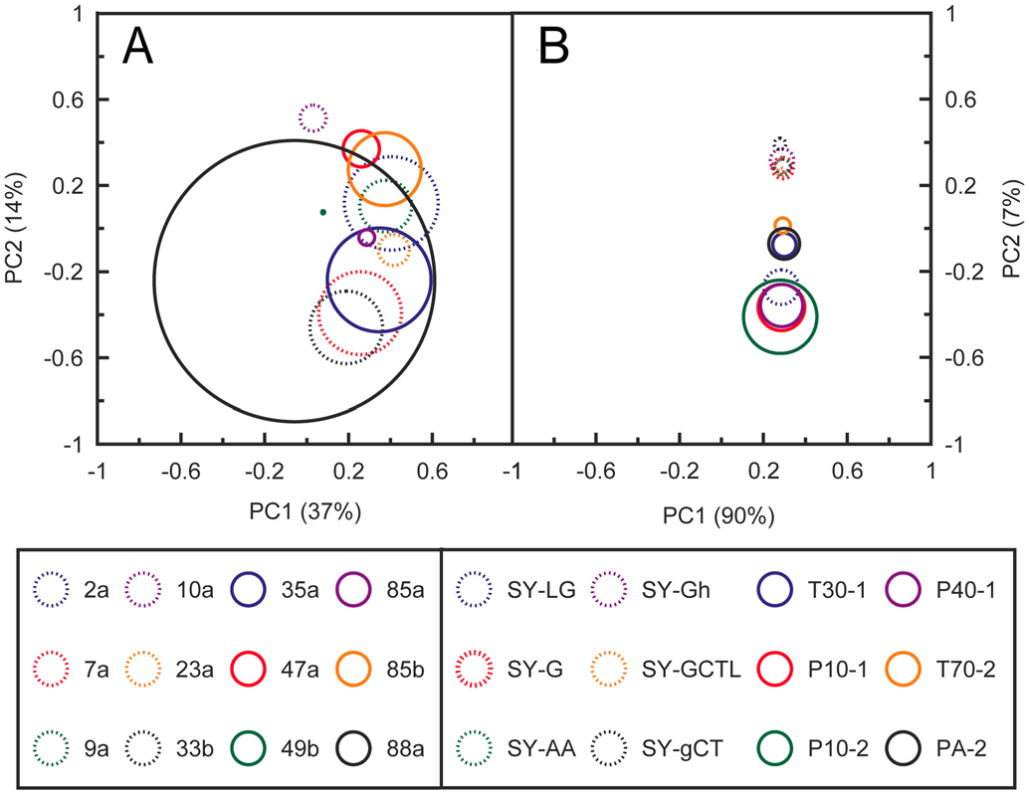
A cartoon to illustrate the relative coverage and overlap of dORs (A) and MOx (B) sensors in odorant space. Principal component analyses were performed separately for the responses of MOx sensors and dORs to 1/100 dilutions of the 110 test compounds. The panels depict the locations of (A) the twelve representative dORs (asterisked in Table S4) and (B) the 12 MOx sensors, in the first two principal components of the loading plots from the PCAs. In each panel the relative half-widths of the tuning curves are used as the radii to generate circles centred on the respective receptors/sensors.

An ideal sensor array for volatile compounds would sample all points in odorant space, using multiple independent sensors. We used a graphical approach to compare, qualitatively, the degree to which the coverage, overlap and independence of dORs and MOx sensors approaches this ideal. (Figure 7). PCAs were run separately for the responses of MOx sensors and 12 of the dORs to 1/100 dilutions of the 110 test compounds. In each case the relative half-widths of the tuning curves were used as the radii to generate circles centred on the location of the respective receptors/sensors in the PCA loading plot. Although this comparison is not strictly quantitative, it illustrates the findings that a set of 12 insect ORs can sample much broader regions of odorant space independently and redundantly than the same number of MOx sensors. The relative effectiveness of the olfactory system of the living fly, which expresses 48 odorant receptors of the seven transmembrane class, rather than the 12 depicted in Figure 7, would be underestimated by this approach.

We also note that the nature of the fly's broad and overlapping sensor fields would seem to require sophisticated and powerful neural processing for classification of odorant signals. There is anatomical, experimental and computational evidence that the insect does have such a system dedicated to olfaction [22], [23]. In contrast, it is known that the chemosensory system of the nematode *Caenorhabditis elegans*, has very few interneurons and synapses between sensory and effector units [24], [25]. Given its deficit in neural processing power, we expect that the nematode approaches one-to-one matching between chemoreceptors and odorants, with less reliance on combinatorial processing than the fly. This would imply that nematode chemoreceptors have very tight tuning curves compared with either fly ORs or MOx sensors and may account for the remarkably large number of chemoreceptor genes identified in the *C. elegans* genome [26]. Whilst replicating the nematode model in an electronic nose may theoretically be feasible, it would present daunting engineering challenges to develop and deploy a very large number of tightly tuned and independent sensors.

### Conclusions

The inferred superior performance of a set of insect odorant receptors over a set of MOx sensors originates with the substantially broader tuning of insect ORs combined with their paradoxically high levels of independence. Differences in sensitivity do not appear to be a critical differentiator between ORs and MOx sensors. However insect ORs appear to have a broader range of sensitivities, which is a corollary of their combination of tuning breadth and independence. The protein structural basis of the unusual ligand-binding characteristics of ORs is unknown and may involve novel mechanisms. Quantifying the gap between MOx sensors and ORs may help inspire and guide new approaches in electronic nose sensor development. From a practical point of view, we believe the benchmarking approach developed here may be used to optimise existing MOx sensor arrays, assess other classes of engineered sensors and possibly to help select suitable applications for them.

## Materials and Methods

### OR and technical sensor data

#### Drosophila antennal data

The responses of 24 antennal receptors to a diverse panel of odorants using the empty neuron system were taken from Hallem et al. [7]. The selected panel of 110 odorants represents a broad sampling of ecologically relevant odours. These include esters, alcohols, ketones, lactones, aldehydes, terpenes, organic acids, amines, sulphur compounds, and aromatics (Table S1).

#### Electronic nose measurements and data processing

One mL dilutions of the 110 volatile organic compounds (VOC) listed in Hallem et al. [7], were prepared in either paraffin oil or water in 20-ml glass vials. Dilutions were chosen to bring the responses for each chemical class into the dynamic range of the sensors, using the following factors: amines, 0.02; lactones, 0.6; acids, 0.08; sulphide/sulphydryl, 0.002; terpenes, 0.2; aldehydes, 0.0001; ketones, 0.0005; aromatics, 0.5; alcohols, 0.0001 and esters, 0.002. There were three exceptions to this scheme, with the dilution factors for methanol and ethanol being 0.003 and, for acetaldehyde, 0.0005. The following odorants were diluted in water: ammonium hydroxide, putrescine, cadaverine, methanoic acid, acetic acid, propionic acid, butyric acid, pentanoic acid, hexanoic acid, isobutyric acid, isopentanoic acid, pyruvic acid, lactic acid, acetaldehyde, methanol and ethanol. All other odorants were diluted in paraffin oil. The vials were capped with silicon/Teflon magnetic autosampler vial caps.

Fox 3000 measurements were made essentially as described previously [4]. Vials were loaded into an autosampler (HS50, CTC Analytics, Switzerland) interfaced with a FOX 3000 E-Nose (Alpha M.O.S, Toulouse, France), which has an array of 12 semiconducting MOx sensors. To equilibrate volatiles with the headspace, samples were incubated at 40° C with shaking (500 rpm) for two minutes. After incubation, 500 µL of the headspace was injected into the FOX 3000 at a rate of 500 µL s^−1^. Dry zero grade air (flow rate 150 mL min^−1^) was used to sweep the sample through the two sensor chambers. A five minute purge was used between samples to allow the sensors to return to baseline. Data were captured and pre-analyzed using AlphaSoft v.11 (Toulouse, France). To simplify data processing, only the maximum resistance changes of each sensor were used for analysis. The computation used for feature extraction is defined as a fractional baseline manipulation given in Eq. (1) below. 
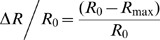
(1)


Where *R*_0_ corresponds to the value of the resistance at *t* = 0 (baseline) and *R*_max_ to the extreme resistance value change when an injection is made. Responses to all odorants were measured independently three times at the same level of humidity and temperature.

### Sensor tuning curves

Mean E-Nose responses were scaled to the same dilution factor, 0.01, as used by Hallem et al. [7] (Table S1). In order to compare the shapes of the tuning curves between ORs and MOx sensors, the data were normalised separately for each individual MOx sensor and odorant receptor. The maximum response in each case was set to unity and data were sorted in descending order. The normalisation focuses attention on the shapes of the curves, rather than their amplitudes.

The half width of each tuning curve was calculated and expressed as the rank number of the odorant giving ≤0.5× the maximum response/110×100. Half widths, ranked in ascending order for Drosophila receptors and MOx sensors are listed in Table S4.

### Independence of sensor responses

The loading plots from a principal component analysis (PCA) were employed to evaluate the independence of the sensors [13]. The scaled responses of the MOx sensors and the responses of dORs to the 110 test compounds were submitted for PCA analysis using Unscrambler software (version 9.1, CAMO PROCESS AS, Nedre Vollgate, Norway) and the loading scores were graphed. (PCA is a technique for calculating the major dimensions of variation for a set of data points in a high dimensional space. It is used when no hypothesis has been formulated as to which dimensions constitute the most relevant information. Principal components are obtained through a linear combination of the dependent variables that maximises the variance within the sample set. The first principal component accounts for the largest quantum of variance among samples. Subsequent principal components account for successive amounts of the total variance in the data set and are uncorrelated with prior principal components). SAS statistical software (Version 9.1, SAS Institute Inc., Cary, NC, USA) was used to calculate Pearson's paired correlation coefficients between sensor-sensor and receptor-receptor pairs.

### Independence of sensors in an ester-specific sub-region of odorant space

Twenty-one esters, with published vapour pressures were selected for a further PCA comparing the 12 MOx sensors and, to ensure an equivalent comparison, a representative set of 12 dORs (Table S4). The 12 Drosophila receptors were chosen by ranking all 24 receptors according to the half-widths of their tuning curves and removing every second receptor from the analysis (Table S4). For MOx sensors, responses were scaled to a 1/100 dilution and corrected for differences in vapour pressure by dividing the sensor responses by the vapour pressures (Table S3). Similarly Hallem's data were corrected by dividing the mean spike rate by the vapour pressures of the compounds in question.

### Compound discrimination

In order to identify the basis of compound discrimination by the two types of sensor arrays we compared the responses of Drosophila receptors and MOx sensors to 25 volatile organic compounds representing five different chemical classes (acids, terpenes, aldehydes, alcohols and esters). For MOx sensor analysis, the headspace concentration of each chemical class was fixed and the volume to be added to a 20 ml glass vial was calculated based on general gas laws. The concentrations used for acids, terpenes, aldehydes, alcohols and esters were 2.0×10^−9^ M, 4.30×10^−10^ M, 4.20×10^−8^ M, 1.40×10^−10^ M and 5.00×10^−9^ M, respectively. These test concentrations were chosen to ensure that compounds from each chemical class elicited responses within the normal dynamic range of the sensors, avoiding saturation and damage to the sensors. The concentrations used were well below the EC_50_ values for many of the test compounds. Responses were normalised to a constant concentration of 4.20×10^−8^ M prior to cluster analysis. Hallem's response data from the same 12 sensors as selected above, for the same compounds were corrected for variations in vapour pressure as described above. It was impossible to determine absolute concentrations for these data. Cluster analysis by Wards' hierarchical clustering technique was performed using SAS statistical software (Version 9.1, SAS Institute Inc., Cary, NC, USA). Radar plots of the same data were used to compare the differentiation of compounds within each of the five functional groups mentioned above. Only results for alcohols are shown (Fig. 4).

### Log-absolute concentration-response characteristics

The absolute sensitivity of the dOR35a Drosophila odorant receptor and two MOx sensors PA/2 and SY/Gh were evaluated for the two test compounds 1-octanol and 1-octen-3-ol.

#### Electronic nose measurements

Dilution series of test compounds were prepared in 1000 µL of paraffin oil in 20 mL autosamper vials so as to generate the desired headspace concentrations. For 1-octanol the headspace concentrations ranged from 2.17×10^−9^ M to 6.97×10^−6^ M and, for 1-octen-3-ol, from 1.28 10×^−9^ M to 3.84×10^−5^ M. Vials were capped with silicon/Teflon magnetic autosampler vials caps and E-Nose measurements were performed as described above. All measurements were done in triplicate.

#### Functional expression of dOR35a in Sf9 cells

Absolute sensitivities of dOR35a to 1-octen-3-ol and 1-octanol were determined by functionally expressing the receptor in *Spodoptera frugiperda* Sf9 cells and measuring odorant responses using a calcium-sensitive dye as described by Kiely et al. [18]. Sf9 cells were maintained in Sf900II medium (Invitrogen) according to the manufacturer's instructions. Sf9 cells were transiently transfected with 500 ng pIB-DmOr35a DNA using Escort IV (Sigma) in 12-well plates. Transfected cells were incubated for 48 hours, to allow the expression of the OR, before calcium imaging of responses to ligands. Fluo4 (Invitrogen) was used as calcium indicator. A 1 M stock solution of each odour was made in dimethylsulfoxide (DMSO) and stored at −20°C. Prior to each assay, odorant solutions were diluted from the stock solution to the desired concentration in saline. Fluorescence images were recorded using a TILL Photonics Imago-QE camera and Nikon Eclipse inverted microscope. Images were recorded every 10 s for 50 s following the addition of: saline (negative control), the test ligand and ionomycin to determine the maximum fluorescence. Images were analysed using the TILLvisION imaging system and ΔF was calculated as the ratio of increase in fluorescence over baseline upon the addition of ligand to the increase in fluorescence over baseline following the addition of ionomycin. Data were analysed and curves fitted using Prism v 5.0 (GraphPad Software).

## Supporting Information

Table S1 Fractional changes in resistance of the 12 metal oxide sensors of the Fox 3000 electronic nose to the 110 odorants used by Hallem et al. (7). Odorants were diluted differentially, according to chemical group, in order to bring the responses into the working range of the sensors. All responses were then adjusted to a nominal dilution of 1/100. Equivalent data for the Drosophila ORs were sourced from Table S1 of Hallem et al. (7).
(0.04 MB PDF)

Table S2 Multivariate Pearson pairwise correlations among MOx sensors (A) and Drosophila ORs (B), using all 110 odorants. Bolded values indicate highly correlated pairs.
(0.04 MB PDF)

Table S3 Listing of 42 compounds, with vapour pressures, used for in-depth comparisons. Compounds 22-42 were used to investigate the sub-region of odorant space defined by esters (Fig. 3). Compounds 16-20 were used for comparisons between sensor responses at constant concentration (Fig. 4). Compounds 1-20 & 22-26 were used to investigate clustering of compounds of different chemical classes (Fig. 5). Compounds 20 & 21 were used to compare absolute sensitivies of MOx sensors and dORs (Fig. 6).
(0.02 MB PDF)

Table S4 Drosophila ORs and MOx sensors ranked by the half-widths of their tuning curves. Asterisked receptors were incorporated into the analyses shown in Figures 3–5 & 7.
(0.02 MB PDF)

Table S5 Multivariate Pearson pairwise correlations among MOx sensors (A) and Drosophila ORs (B), within the odorant space defined by compounds 22-42 (i.e. esters) of Table S3 and Figure 3. Bolded values include highly correlated pairs.
(0.05 MB PDF)
