# Supplementary material for: Bio-Benchmarking of Electronic Nose Sensors
Source: PLoS One. 2009 Jul 29;4(7):e6406. doi: 10.1371/journal.pone.0006406 (PMC2712691; doi:10.1371/journal.pone.0006406)
Supplement: Table S1 — Fractional changes in resistance of the 12 metal oxide sensors of the Fox 3000 electronic nose to the 110 odorants used by Hallem et al. (7). Odorants were diluted differentially, according to chemical group, in order to bring the responses into the working range of the sensors. All responses were then adjusted to a nominal dilution of 1/100. Equivalent data for the Drosophila ORs were sourced from Table S1 of Hallem et al. (7). (0.04 MB PDF) [file pone.0006406.s001.pdf]

	compound	group	SY-LG	SY-G	SY-AA	SY-Gh	SY-GCTL	SY-gCT	T30-1	P10-1	P10-2	P40-1	T70-2	PA-2
1	ammonium hydroxide	amine	0.10150	0.35200	0.21000	0.41800	0.03815	0.54833	0.34433	0.41000	0.28700	0.42817	0.34000	0.46633
2	putrescine	amine	0.02235	0.01005	0.00995	0.01007	0.00506	0.01117	0.08283	0.14700	0.12833	0.15400	0.05417	0.11817
3	cadaverine	amine	0.02145	0.00668	0.00908	0.00623	0.00354	0.00682	0.08600	0.14483	0.12750	0.15083	0.05767	0.10667
4	g-butyrolactone	lactone	0.00040	0.00536	0.00203	0.00550	0.00088	0.00464	0.00816	0.00811	0.00449	0.00804	0.00543	0.01168
5	g-hexalactone	lactone	0.00112	0.00319	0.00132	0.00266	0.00252	0.00153	0.00311	0.00271	0.00164	0.00242	0.00194	0.00469
6	g-octalactone	lactone	0.00060	0.00101	0.00052	0.00079	0.00068	0.00049	0.00305	0.00447	0.00384	0.00464	0.00191	0.00384
7	g-decalactone	lactone	0.00028	0.00011	0.00010	0.00011	0.00010	0.00009	0.00058	0.00103	0.00081	0.00111	0.00036	0.00071
8	d-decalactone	lactone	0.00008	0.00011	0.00011	0.00011	0.00003	0.00009	0.00052	0.00067	0.00041	0.00071	0.00032	0.00094
9	methanoic acid	acid	0.00865	0.00898	0.00705	0.00653	0.00776	0.00378	0.02388	0.03725	0.03233	0.03871	0.01563	0.03229
10	acetic acid	acid	0.01084	0.06404	0.03296	0.05083	0.01575	0.03192	0.05521	0.05383	0.04329	0.05746	0.04433	0.07317
11	propionic acid	acid	0.01175	0.06425	0.03625	0.05083	0.02113	0.02979	0.06254	0.06392	0.05367	0.06758	0.04758	0.08925
12	butyric acid	acid	0.01463	0.07783	0.04125	0.06438	0.02721	0.03783	0.07213	0.07113	0.06175	0.07508	0.05529	0.09879
13	pentanoic acid	acid	0.00582	0.02067	0.01379	0.01546	0.00929	0.00693	0.02958	0.03963	0.03721	0.04146	0.01942	0.04746
14	hexanoic acid	acid	0.00443	0.00664	0.00486	0.00500	0.00362	0.00271	0.01971	0.03554	0.03129	0.03671	0.01192	0.02750
15	heptanoic acid	acid	0.00366	0.00245	0.00132	0.00223	0.00192	0.00166	0.01817	0.03267	0.03021	0.03383	0.01125	0.01854
16	octanoic acid	acid	0.00330	0.00170	0.00096	0.00146	0.00137	0.00112	0.01663	0.03196	0.02988	0.03321	0.01018	0.01558
17	nonanoic acid	acid	0.00413	0.00373	0.00232	0.00256	0.00311	0.00163	0.01725	0.03175	0.02938	0.03296	0.01042	0.01713
18	linoleic acid	acid	0.00367	0.00282	0.00165	0.00250	0.00209	0.00193	0.02067	0.03496	0.03075	0.03625	0.01199	0.02917
19	isobutyric acid	acid	0.02479	0.11383	0.06067	0.10383	0.04738	0.07550	0.09300	0.09133	0.07625	0.09533	0.07721	0.11304
20	isopentanoic acid	acid	0.01367	0.05225	0.02938	0.04179	0.02325	0.02283	0.05996	0.06463	0.05625	0.06821	0.04100	0.08958
21	pyruvic acid	acid	0.00448	0.00920	0.00655	0.00721	0.00506	0.00362	0.02338	0.03833	0.03429	0.03963	0.01425	0.03500
22	2-ethylhexanoic acid	acid	0.00403	0.00439	0.00199	0.00330	0.00245	0.00284	0.02133	0.03442	0.02996	0.03538	0.01234	0.02758
23	lactic acid	acid	0.00330	0.00162	0.00117	0.00172	0.00136	0.00130	0.01542	0.03025	0.02796	0.03154	0.00918	0.01450
24	3-methylthio-1-propanol	sulphur	0.25750	0.18450	0.12133	0.13683	0.12983	0.07383	0.41300	0.39500	0.34633	0.42033	0.29533	0.56167
25	dimethyl sulfide	sulphur	1.81167	8.03333	4.71667	7.90000	3.16000	7.95000	4.75167	4.70167	4.36500	4.72167	4.69333	4.92500
26	terpinolene	terpene	0.02658	0.04828	0.02572	0.04663	0.03925	0.03665	0.03167	0.02867	0.02650	0.02993	0.02783	0.04083
27	a -pinene	terpene	0.03260	0.07617	0.05200	0.07833	0.06633	0.06833	0.03978	0.03682	0.03212	0.03798	0.03785	0.04610
28	b -pinene	terpene	0.03225	0.06650	0.04427	0.06733	0.05233	0.05500	0.03685	0.03445	0.02998	0.03560	0.03440	0.04492
29	(1S)-(+)-3-carene	terpene	0.02720	0.06417	0.04093	0.06633	0.05150	0.06033	0.03768	0.03590	0.02977	0.03733	0.03560	0.04498
30	limonene	terpene	0.02682	0.06083	0.04212	0.06267	0.04527	0.05400	0.03458	0.03318	0.02785	0.03438	0.03213	0.04342
31	a-humulene	terpene	0.00236	0.00690	0.00411	0.00618	0.00198	0.00471	0.01360	0.01628	0.01042	0.01747	0.00872	0.02618
32	b-myrcene	terpene	0.02080	0.06433	0.04173	0.06450	0.05178	0.05203	0.03705	0.03307	0.02965	0.03408	0.03547	0.04417
33	(-)-trans-caryophyllene	terpene	0.00657	0.00967	0.00587	0.00847	0.00392	0.00633	0.02120	0.02128	0.01305	0.02302	0.01675	0.03273
34	p-cymene	terpene	0.01102	0.04390	0.02548	0.04037	0.01828	0.03467	0.03247	0.03157	0.02228	0.03283	0.02788	0.04282
35	geranyl acetate	terpene	0.00469	0.01843	0.01087	0.01788	0.00465	0.01495	0.02645	0.02877	0.01863	0.03052	0.02045	0.03910
36	a -terpineol	terpene	0.02997	0.04935	0.02632	0.05130	0.04330	0.03937	0.03045	0.02852	0.02607	0.02917	0.02697	0.04053
37	geraniol	terpene	0.00367	0.02427	0.01342	0.02777	0.00447	0.03172	0.03267	0.03342	0.02200	0.03547	0.02747	0.04237
38	nerol	terpene	0.00217	0.00627	0.00399	0.00552	0.00170	0.00460	0.01672	0.01832	0.01222	0.01957	0.01142	0.02805
39	linalool	terpene	0.01025	0.01430	0.00847	0.01322	0.01030	0.00778	0.01917	0.01682	0.01312	0.01823	0.01590	0.02915
40	b-citronellol	terpene	0.00216	0.00338	0.00216	0.00291	0.00138	0.00206	0.00838	0.00948	0.00642	0.01020	0.00485	0.01708
41	linalool oxide	terpene	0.01158	0.01753	0.00957	0.01733	0.01182	0.01185	0.01987	0.01828	0.01637	0.01913	0.01657	0.02928
42	acetaldehyde	aldehyde	0.52000	8.66000	6.52667	8.08667	2.73333	7.85333	14.74000	12.48667	8.51333	13.34000	13.66667	17.15333
43	propanal	aldehyde	7.31333	53.33333	28.63333	45.76667	15.23333	40.03333	60.40000	57.33333	46.83333	60.50000	45.06667	81.86667
44	butanal	aldehyde	6.97000	49.10000	24.96667	40.30000	14.80000	32.53333	51.83333	52.96667	45.56667	55.43333	34.23333	76.90000
45	pentanal	aldehyde	4.88000	18.26667	8.76667	13.03333	6.60333	8.86667	26.96667	35.46667	32.20000	36.66667	15.43333	44.83333
46	hexanal	aldehyde	3.93000	8.69667	3.97333	6.05000	3.64333	3.79000	18.43333	30.20000	27.63333	31.13333	10.63000	27.93333
47	E2-hexenal	aldehyde	3.48000	6.39667	3.09667	4.44000	2.76333	2.76000	16.83333	29.26667	26.56667	30.13333	10.29000	23.93333
48	furfural	aldehyde	2.75333	3.74333	1.62667	2.75667	0.60600	1.90333	14.80000	27.96667	24.36667	28.90000	8.83000	21.80000
49	2-propenal	aldehyde	3.67333	30.73333	22.00000	23.93333	5.14333	19.46667	65.70000	57.70000	37.00000	61.26667	55.16667	83.86667
50	acetone	ketone	1.65733	13.66667	5.43333	16.64667	2.29333	19.06000	16.25333	15.73333	12.08667	16.38667	14.22000	18.84667
51	2-butanone	ketone	1.96800	15.27333	6.30667	16.08000	3.53333	16.20000	14.70667	14.54667	11.56667	15.17333	11.76667	18.36667
52	2-pentanone	ketone	1.79200	12.68000	5.06000	12.61333	3.14667	11.39333	12.99333	13.24667	10.70667	13.78667	9.48000	17.61333
53	2-heptanone	ketone	0.80333	1.95800	0.74067	1.46467	0.70000	0.89600	4.21333	6.48000	5.97333	6.69333	2.26667	7.06667
54	6-methyl-5-hepten-2-one	ketone	0.62867	0.77533	0.42467	0.54467	0.47667	0.29133	2.80000	5.64000	4.98000	5.82000	1.63867	3.79333
55	2,3-butanedione	ketone	3.38667	25.13333	12.59333	22.93333	7.38000	18.47333	13.20667	12.62000	9.76667	13.24000	10.12000	17.32667
56	phenethyl alcohol	aromatic	0.00044	0.00046	0.00038	0.00040	0.00016	0.00023	0.00017	0.00062	0.00026	0.00063	0.00005	0.00051
57	benzyl alcohol	aromatic	0.00082	0.00179	0.00104	0.00135	0.00051	0.00070	0.00111	0.00115	0.00059	0.00115	0.00046	0.00313
58	methyl salicylate	aromatic	0.00208	0.00210	0.00139	0.00150	0.00144	0.00061	0.00281	0.00331	0.00264	0.00355	0.00198	0.00455
59	methyl benzoate	aromatic	0.00062	0.00296	0.00113	0.00245	0.00090	0.00181	0.00814	0.00741	0.00504	0.00793	0.00641	0.01329
60	ethyl benzoate	aromatic	0.00033	0.00486	0.00242	0.00409	0.00119	0.00343	0.00936	0.00851	0.00611	0.00914	0.00711	0.01339
61	phenethyl acetate	aromatic	0.00012	0.00079	0.00048	0.00067	0.00031	0.00052	0.00258	0.00260	0.00172	0.00281	0.00144	0.00523
62	benzaldehyde	aromatic	0.00141	0.02640	0.01442	0.02433	0.00727	0.02107	0.01069	0.01121	0.00624	0.01175	0.00795	0.01715
63	phenylacetaldehyde	aromatic	0.00213	0.01171	0.00578	0.01111	0.00560	0.00701	0.00879	0.00721	0.00643	0.00808	0.00801	0.01235
64	acetophenone	aromatic	0.00089	0.01225	0.00535	0.01153	0.00403	0.00799	0.00645	0.00560	0.00436	0.00473	0.00433	0.01288
65	ethyl cinnamate	aromatic	0.00025	0.00042	0.00017	0.00038	0.00007	0.00030	0.00203	0.00202	0.00159	0.00220	0.00141	0.00329
66	2-methylphenol	aromatic	0.00425	0.00691	0.00342	0.00625	0.00587	0.00342	0.00574	0.00344	0.00330	0.00293	0.00413	0.00735
67	4-ethyl guaiacol	aromatic	0.00036	0.00237	0.00100	0.00205	0.00054	0.00172	0.00969	0.00879	0.00697	0.00938	0.00783	0.01293
68	eugenol	aromatic	0.00014	0.00094	0.00056	0.00087	0.00011	0.00080	0.00409	0.00379	0.00203	0.00395	0.00336	0.00593
69	methanol	alcohol	0.18789	1.25667	0.54667	1.12333	0.18356	1.01111	2.63111	2.20778	1.72444	2.33889	2.52667	2.91889
70	ethanol	alcohol	0.19278	1.41222	0.64333	1.30444	0.23889	1.08778	2.18222	2.10000	1.63222	2.21000	1.76556	2.75222
71	1-propanol	alcohol	5.13667	41.20000	17.90000	36.83333	7.57000	28.36667	50.63333	54.16667	41.93333	56.63333	31.00000	73.90000
72	1-butanol	alcohol	4.56667	32.06667	13.80000	27.13333	6.84667	18.13333	39.46667	46.26667	37.50000	47.90000	21.96667	61.60000
73	1-pentanol	alcohol	3.22000	8.24000	3.45333	6.54000	2.42333	3.95667	18.56667	30.90000	27.23333	31.83333	10.22667	27.63333
74	1-hexanol	alcohol	2.69667	3.22333	1.47667	2.60000	1.17000	1.57667	13.33333	27.66667	24.66667	28.40000	7.72000	16.63333
75	1-octanol	alcohol	2.57000	0.69467	0.44267	0.84267	0.14200	0.76433	11.23333	25.43333	22.36667	26.16667	6.33000	11.90000
76	2-pentanol	alcohol	3.98333	13.96667	5.19333	12.63333	2.69333	7.85000	30.90000	39.60000	32.03333	40.66667	16.40000	49.20000
77	3-methylbutanol	alcohol	3.61000	12.50000	5.37667	9.53667	3.53333	5.32667	24.23333	34.40000	30.23333	35.40000	12.70000	37.46667
78	3-methyl-2-buten-1-ol	alcohol	3.23000	5.70000	2.77333	4.32333	1.33000	2.63000	15.80000	28.73333	25.30000	29.63333	9.13667	21.70000
79	1-penten-3-ol	alcohol	6.27333	24.46667	10.63333	20.16667	7.22000	12.33333	34.90000	40.16667	32.66667	41.33333	21.53333	49.40000
80	1-octen-3-ol	alcohol	2.43667	1.16333	0.48733	1.18667	0.58867	0.63767	9.75667	22.60000	19.50000	23.36667	5.40000	10.56667
81	E2-hexenol	alcohol	2.80000	2.74000	1.31667	2.22000	1.04467	1.09833	12.56667	26.73333	23.73333	27.50000	7.59000	15.30000
82	Z2-hexenol	alcohol	2.90667	2.90667	1.75667	2.40333	1.35333	1.25000	12.76667	26.86667	23.90000	27.66667	7.83000	15.56667
83	E3-hexenol	alcohol	3.16667	4.31000	2.46333	3.35333	2.21667	1.84000	13.83333	27.36667	24.43333	28.20000	8.53000	17.50000
84	Z3-hexenol	alcohol	3.09667	3.48333	2.04000	2.74667	1.73667	1.61000	13.20000	27.10000	24.10000	27.93333	8.06000	16.23333
85	glycerol	alcohol	2.70667	0.76367	0.41967	0.98933	0.14300	0.81833	11.30000	25.60000	22.56667	26.40000	6.51667	12.23333
86	2,3-butanediol	alcohol	2.55333	0.68700	0.59167	1.03767	0.20700	0.74167	10.73333	24.66667	21.70000	25.40000	6.18333	11.63333
87	methyl acetate	ester	0.52867	1.69500	0.37400	2.47333	0.27500	2.30000	4.75000	4.55333	3.89500	4.65667	4.62333	4.91167
88	ethyl acetate	ester	0.38500	1.44500	0.32567	1.73167	0.21817	1.32833	4.32000	4.18000	3.37667	4.33500	3.86000	4.78000
89	propyl acetate	ester	0.26833	1.23500	0.35133	1.21000	0.24633	0.81000	3.43833	3.34000	2.29333	3.52167	2.61000	4.37333
90	butyl acetate	ester	0.20300	0.98000	0.22600	0.86750	0.19350	0.53000	2.62750	2.59750	1.66750	2.73500	1.72000	3.81250
91	pentyl acetate	ester	0.11833	0.50433	0.19250	0.38650	0.13633	0.19150	1.12000	1.17167	0.86167	1.23167	0.60333	2.09000
92	hexyl acetate	ester	0.04985	0.20375	0.05625	0.15075	0.04298	0.05168	0.40925	0.52750	0.44700	0.55500	0.20075	0.84000
93	isobutyl acetate	ester	0.23817	0.98000	0.20383	0.79667	0.36617	0.42950	3.00167	2.75667	1.88500	2.90667	2.14833	3.97167
94	isopentyl acetate	ester	0.14233	0.59000	0.13450	0.42950	0.14567	0.20483	1.49667	1.52833	1.11667	1.59333	0.83333	2.60167
95	E2-hexenyl acetate	ester	0.29217	1.16333	0.73167	0.90500	0.43367	0.44483	1.27167	1.02667	0.80667	1.12333	0.97167	1.94833
96	methyl butyrate	ester	0.54417	1.48167	0.45950	1.38667	0.37767	0.99667	3.97167	3.62000	2.87000	3.81667	3.44000	4.49667
97	ethyl butyrate	ester	0.33183	0.76667	0.19067	0.65000	0.21733	0.38200	2.56000	2.54833	1.95500	2.70833	1.67833	3.67333
98	hexyl butyrate	ester	0.05102	0.06362	0.02710	0.05415	0.01275	0.02690	0.22517	0.44933	0.33500	0.48083	0.09383	0.45017
99	ethyl 3-hydroxybutyrate	ester	0.31500	0.84667	0.56467	0.62517	0.31967	0.26433	1.12500	0.91167	0.82500	0.97667	0.77500	1.85167
100	ethyl propionate	ester	0.41550	1.40833	0.41950	1.38000	0.26017	0.97500	3.84167	3.62833	2.81833	3.82333	3.17167	4.53000
101	ethyl methanoate	ester	0.39433	3.17000	0.92500	4.31667	0.54167	4.95667	4.67000	4.46167	3.90833	4.56833	4.48333	4.88833
102	methyl hexanoate	ester	0.18517	0.35967	0.08917	0.27833	0.13367	0.14950	1.00500	1.07333	0.90667	1.13500	0.47867	1.88167
103	ethyl hexanoate	ester	0.09850	0.20417	0.05145	0.14350	0.08350	0.07700	0.41550	0.61833	0.55883	0.65167	0.16083	0.90833
104	hexyl hexanoate	ester	0.04977	0.01560	0.01560	0.02002	0.00407	0.02055	0.14683	0.38200	0.28400	0.40550	0.05983	0.23767
105	methyl octanoate	ester	0.05067	0.03708	0.01723	0.03420	0.01025	0.02058	0.17600	0.38533	0.30067	0.40900	0.08467	0.28100
106	ethyl octanoate	ester	0.04732	0.01970	0.01810	0.02010	0.00212	0.01672	0.16900	0.36967	0.28050	0.39167	0.08467	0.25483
107	ethyl decanoate	ester	0.04587	0.02593	0.01660	0.02218	0.00493	0.01677	0.18317	0.37767	0.29667	0.40100	0.09917	0.27733
108	ethyl trans-2-butenoate	ester	0.32367	1.14667	0.25717	0.95167	0.18317	0.59333	2.20333	1.96333	1.45167	2.09000	1.48667	3.20167
109	ethyl lactate	ester	0.82500	4.32000	1.96833	3.82500	1.35667	2.53667	3.32833	2.67000	2.10167	2.84500	2.81167	4.05000
110	diethyl succinate	ester	0.03717	0.01075	0.00988	0.02457	0.00183	0.02813	0.12300	0.37633	0.27700	0.40167	0.02877	0.19667
