# Supplementary material for: Bio-Benchmarking of Electronic Nose Sensors
Source: PLoS One. 2009 Jul 29;4(7):e6406. doi: 10.1371/journal.pone.0006406 (PMC2712691; doi:10.1371/journal.pone.0006406)
Supplement: Table S2 — Multivariate Pearson pairwise correlations among MOx sensors (A) and Drosophila ORs (B), using all 110 odorants. Bolded values indicate highly correlated pairs. (0.04 MB PDF) [file pone.0006406.s002.pdf]

A

SY-LG	SY-G	SY-AA	SY-Gh	SY-GCTL	SY-gCT	T30-1	P10-1	P10-2	P40-1	T70-2	PA-2	
1.00	0.84	0.81	0.82	0.87	0.77	0.91	0.96	0.97	0.96	0.84	0.91	SY-LG
	1.00	0.98	1.00	0.97	0.97	0.93	0.82	0.79	0.82	0.91	0.93	SY-G
		1.00	0.97	0.96	0.96	0.93	0.81	0.77	0.81	0.94	0.93	SY-AA
			1.00	0.96	0.99	0.91	0.80	0.77	0.80	0.90	0.91	SY-Gh
				1.00	0.94	0.88	0.79	0.78	0.80	0.86	0.89	SY-GCTL
					1.00	0.87	0.74	0.71	0.75	0.88	0.87	SY-gCT
						1.00	0.95	0.92	0.95	0.98	0.99	T30-1
							1.00	0.99	1.00	0.90	0.95	P10-1
								1.00	0.99	0.86	0.92	P10-2
									1.00	0.90	0.95	P40-1
										1.00	0.97	T70-2
											1.00	PA-2

B

2a	7a	9a	10a	19a	22a	23a	33b	35a	43a	43b	47a	47b	49b	59b	65a	67a	67c	82a	85a	85b	85f	88a	98a	
**1.00**	0.37	0.67	0.10	0.50	0.63	**0.72**	0.29	0.48	0.46	0.58	0.57	-0.57	0.02	0.19	0.50	0.42	0.60	0.38	0.38	**0.72**	0.62	-0.09	0.59	**2a**
	**1.00**	0.27	-0.10	0.19	0.13	0.52	0.35	0.56	0.33	0.20	-0.01	-0.25	0.13	0.07	-0.03	0.19	0.40	0.16	0.22	0.21	0.39	-0.06	0.07	**7a**
		**1.00**	0.22	0.34	0.53	0.63	0.40	0.43	0.54	**0.74**	0.48	-0.53	0.18	0.30	0.24	0.35	0.63	0.32	0.47	0.53	**0.72**	-0.07	0.41	**9a**
			**1.00**	-0.09	0.20	-0.03	-0.17	-0.15	0.04	0.23	0.21	-0.03	0.16	0.00	0.00	0.32	0.03	-0.03	-0.01	0.09	0.32	-0.11	0.44	**10a**
				**1.00**	0.32	0.60	0.19	0.46	0.61	0.29	0.36	-0.21	0.03	-0.04	0.30	0.37	0.38	0.33	0.33	0.48	0.31	0.12	0.47	**19a**
					**1.00**	0.44	0.23	0.31	0.23	0.61	**0.71**	-0.59	-0.02	0.21	0.47	0.36	0.33	0.25	0.17	0.54	0.37	-0.09	0.53	**22a**
						**1.00**	0.33	0.64	0.69	0.56	0.33	-0.40	0.13	0.15	0.27	0.44	0.64	0.42	0.49	0.68	0.64	-0.05	0.43	**23a**
							**1.00**	0.30	0.24	0.38	0.00	-0.24	0.01	0.20	-0.11	0.00	0.43	0.24	0.22	0.01	0.16	0.17	0.01	**33b**
								**1.00**	0.51	0.35	0.32	-0.41	0.10	0.07	0.21	0.37	0.45	0.28	0.40	0.46	0.49	-0.03	0.28	**35a**
									**1.00**	0.44	0.26	-0.36	0.54	0.07	0.07	0.40	0.55	0.29	0.45	0.47	0.61	0.00	0.30	**43a**
										**1.00**	0.53	-0.41	0.10	0.37	0.15	0.26	0.61	0.28	0.49	0.54	0.54	-0.04	0.47	**43b**
											**1.00**	-0.44	0.00	0.24	0.51	0.36	0.23	0.21	0.13	0.56	0.38	0.10	0.55	**47a**
												**1.00**	-0.19	-0.20	-0.29	-0.20	-0.44	-0.22	-0.27	-0.40	-0.47	0.23	-0.31	**47b**
													**1.00**	-0.04	-0.11	0.30	0.21	-0.01	0.12	0.06	0.36	-0.12	0.02	**49b**
														**1.00**	0.10	0.02	0.32	0.19	0.16	0.07	0.26	0.14	0.01	**59b**
															**1.00**	0.28	0.18	0.16	0.17	0.51	0.30	-0.09	0.46	**65a**
																**1.00**	0.27	0.25	0.23	0.42	0.50	-0.08	0.55	**67a**
																	**1.00**	0.32	0.51	0.43	0.69	-0.03	0.30	**67c**
																		**1.00**	0.16	0.22	0.25	0.02	0.32	**82a**
																			**1.00**	0.46	0.44	-0.14	0.32	**85a**
																				**1.00**	0.60	-0.26	0.66	**85b**
																					**1.00**	-0.25	0.44	**85f**
																						**1.00**	-0.08	**88a**
																							**1.00**	**98a**
