# Supplementary material for: Bio-Benchmarking of Electronic Nose Sensors
Source: PLoS One. 2009 Jul 29;4(7):e6406. doi: 10.1371/journal.pone.0006406 (PMC2712691; doi:10.1371/journal.pone.0006406)
Supplement: Table S3 — Listing of 42 compounds, with vapour pressures, used for in-depth comparisons. Compounds 22-42 were used to investigate the sub-region of odorant space defined by esters (Fig. 3). Compounds 16-20 were used for comparisons between sensor responses at constant concentration (Fig. 4). Compounds 1-20 & 22-26 were used to investigate clustering of compounds of different chemical classes (Fig. 5). Compounds 20 & 21 were used to compare absolute sensitivies of MOx sensors and dORs (Fig. 6). (0.02 MB PDF) [file pone.0006406.s003.pdf]

	Compound	chemical class	Vapour Pressure (Pa)
1	acetic acid	acid	1.37E+04
2	butyric acid	acid	1.02E+03
3	heptanoic acid	acid	1.19E+02
4	octanoic acid	acid	2.43E+01
5	isopentanoic acid	acid	4.56E+02
6	geranyl acetate	terpene	8.51E+01
7	geraniol	terpene	2.74E+00
8	nerol	terpene	2.43E+01
9	linalool	terpene	3.17E+02
10	b-citronellol	terpene	2.43E+01
11	acetaldehyde	aldehyde	8.92E+05
12	butanal	aldehyde	1.09E+05
13	hexanal	aldehyde	1.21E+04
14	E2-hexenal	aldehyde	6.08E+03
15	furfural	aldehyde	2.46E+03
16	1-pentanol	alcohol	3.37E+03
17	1-hexanol	alcohol	6.08E+02
18	3-methylbutanol	alcohol	3.60E+03
19	Z2-hexenol	alcohol	1.21E+04
20	1-octen-3-ol	alcohol	1.00E+03
21	1-octanol	alcohol	1.96E+01
22	methyl acetate	ester 1	2.07E+05
23	ethyl acetate	ester 1	1.36E+05
24	isopentyl acetate	ester 1	4.83E+03
25	ethyl butyrate	ester 1	1.29E+04
26	ethyl hexanoate	ester 1	2.02E+03
27	propyl acetate	ester 2	4.28E+04
28	butyl acetate	ester 2	9.73E+03
29	pentyl acetate	ester 2	4.86E+03
30	hexyl acetate	ester 2	1.69E+03
31	isobutyl acetate	ester 2	2.43E+04
32	methyl butyrate	ester 2	4.85E+04
33	hexyl butyrate	ester 2	2.83E+02
34	ethyl 3-hydroxybutyrate	ester 2	1.02E+03
35	ethyl propionate	ester 2	4.85E+04
36	ethyl methanoate	ester 2	2.38E+05
37	methyl hexanoate	ester 2	4.80E+03
38	methyl octanoate	ester 2	6.08E+03
39	ethyl octanoate	ester 2	2.46E+02
40	ethyl decanoate	ester 2	1.82E+02
41	ethyl lactate	ester 2	2.46E+03
42	diethyl succinate	ester 2	3.65E+02
