# Supplementary material for: Bio-Benchmarking of Electronic Nose Sensors
Source: PLoS One. 2009 Jul 29;4(7):e6406. doi: 10.1371/journal.pone.0006406 (PMC2712691; doi:10.1371/journal.pone.0006406)
Supplement: Table S4 — Drosophila ORs and MOx sensors ranked by the half-widths of their tuning curves. Asterisked receptors were incorporated into the analyses shown in Figures 3– 5 & 7. (0.02 MB PDF) [file pone.0006406.s004.pdf]

**Drosophila OR sensor**	**Half width of tuning curve**	**Rank order**	**MOx sensor**	**Half width of tuning curve**
Or82a	0.7%	1	SY/gCTL*	1.8%
Or49b*	0.7%	2	SY/gCT*	2.7%
Or67c	2.4%	3	SY/AA*	3.6%
Or85a*	3.7%	4	T70/2*	3.6%
Or59b	4.9%	5	SY/G*	4.6%
Or10a*	6.1%	6	SY/GH*	5.5%
Or65a	7.3%	7	T30/1*	5.5%
Or23a*	7.3%	8	PA/2*	7.3%
Or43a	7.3%	9	SY/LG*	8.2%
Or47a*	8.5%	10	P40/1*	10.0%
Or43b	12.2%	11	P10/1*	10.9%
Or9a*	12.2%	12	P10/2*	17.3%
Or22a	14.6%	13		
Or33b*	17.1%	14		
Or98a	17.1%	15		
Or85b*	17.1%	16		
Or85f	19.5%	17		
Or7a*	19.5%	18		
Or19a	20.7%	19		
Or2a*	22.0%	20		
Or67a	23.2%	21		
Or35a*	24.4%	22		
Or47b	36.6%	23		
Or88a*	65.9%	24		
