# Supplementary material for: Bio-Benchmarking of Electronic Nose Sensors
Source: PLoS One. 2009 Jul 29;4(7):e6406. doi: 10.1371/journal.pone.0006406 (PMC2712691; doi:10.1371/journal.pone.0006406)
Supplement: Table S5 — Multivariate Pearson pairwise correlations among MOx sensors (A) and Drosophila ORs (B), within the odorant space defined by compounds 22-42 (i.e. esters) of Table S3 and Figure 3. Bolded values include highly correlated pairs. (0.05 MB PDF) [file pone.0006406.s005.pdf]

A

SY/LG	SY/G	SY/AA	SY/Gh	SY/GCTL	SY/gCT	T30/1	P10/1	P10/2	P40/1	T70/2	PA/2	
**1.000**	**0.765**	**0.824**	**0.751**	**0.756**	**0.713**	**0.984**	**0.829**	**0.855**	**0.830**	**0.934**	**0.957**	**SY/LG**
	**1.000**	**0.981**	**0.997**	**0.994**	**0.974**	**0.786**	0.348	0.374	0.349	**0.920**	0.675	**SY/G**
		**1.000**	**0.967**	**0.989**	**0.923**	**0.826**	0.392	0.426	0.394	**0.940**	**0.731**	**SY/AA**
			**1.000**	**0.985**	**0.989**	**0.774**	0.352	0.374	0.353	**0.909**	0.653	**SY/Gh**
				**1.000**	**0.951**	**0.768**	0.306	0.338	0.307	**0.908**	0.661	**SY/GCTL**
					**1.000**	**0.737**	0.352	0.366	0.353	**0.877**	0.599	**SY/gCT**
						**1.000**	**0.826**	**0.847**	**0.826**	**0.954**	**0.973**	**T30/1**
							**1.000**	**0.997**	**1.000**	0.638	**0.858**	**P10/1**
								**1.000**	**0.998**	0.665	**0.879**	**P10/2**
									**1.000**	0.639	**0.859**	**P40/1**
										**1.000**	**0.874**	**T70/2**
											**1.000**	**PA/2**

B

2a	7a	9a	10a	23a	33b	35a	47a	49b	85a	85b	88a	
**1.00**	-0.62	**0.82**	0.36	**0.75**	-0.23	0.08	0.55	0.43	0.35	0.58	-0.28	**2a**
	**1.00**	-0.31	-0.63	-0.23	0.16	-0.02	-0.23	0.23	0.16	-0.59	0.58	**7a**
		**1.00**	0.19	0.58	-0.14	0.02	0.43	**0.76**	0.66	0.45	-0.26	**9a**
			**1.00**	-0.10	0.38	-0.09	-0.37	0.15	-0.17	0.24	-0.52	**10a**
				**1.00**	-0.49	-0.03	0.47	0.31	0.24	0.21	0.31	**23a**
					**1.00**	0.55	-0.32	0.31	0.30	0.12	-0.33	**33b**
						**1.00**	0.25	0.12	0.60	0.69	-0.43	**35a**
							**1.00**	0.03	0.29	0.41	-0.03	**47a**
								**1.00**	**0.76**	0.13	-0.07	**49b**
									**1.00**	0.54	-0.31	**85a**
										**1.00**	**-0.79**	**85b**
											**1.00**	**88a**
